# Perceptions of flatulence from bean consumption among adults in 3 feeding studies

**DOI:** 10.1186/1475-2891-10-128

**Published:** 2011-11-21

**Authors:** Donna M Winham, Andrea M Hutchins

**Affiliations:** 1 Nutrition Program, School of Nutrition and Health Promotion, Arizona State University, 500 North 3rd Street, Phoenix, AZ 85004 USA; 2 Department of Health Sciences, University of Colorado Colorado Springs, 1420 Austin Bluffs Parkway, Colorado Springs, CO 80918 USA

**Keywords:** legumes, beans, gastrointestinal symptoms, flatulence, food perceptions, dietary fiber

## Abstract

**Background:**

Many consumers avoid eating beans because they believe legume consumption will cause excessive intestinal gas or flatulence. An increasing body of research and the 2010 Dietary Guidelines for Americans supports the benefits of a plant-based diet, and legumes specifically, in the reduction of chronic disease risks. The purpose of the current research was to investigate the perception of increased flatulence and gastrointestinal discomfort among participants who consumed a ½ cup of beans daily for 8 or 12 weeks.

**Methods:**

Participants in three studies to test the effects of beans on heart disease biomarkers completed the same weekly questionnaire to assess gastrointestinal discomfort issues such as increased flatulence, stool changes, and bloating. Studies 1 and 2 were randomized crossover trials. Participants consumed ½ cup of pinto beans, black-eyed peas, and canned carrots as control (n = 17) in Study 1 for three randomized 8-week phases. For Study 2, participants ate ½ cup baked beans or canned carrots as control (n = 29) for two randomized 8-week phases. Study 3 was a parallel arm trial with 40 subjects receiving ½ cup pinto beans and 40 consuming a control soup for 12 weeks. Changes in the frequency of perceived flatulence, stool characteristics, and bloating were the primary outcome measures. Chi-square distributions were examined for the presence or absence of symptoms and demographic characteristics to determine differences by gender, age, body mass index (BMI), and bean type.

**Results:**

Less than 50% reported increased flatulence from eating pinto or baked beans during the first week of each trial, but only 19% had a flatulence increase with black-eyed peas. A small percentage (3-11%) reported increased flatulence across the three studies even on control diets without flatulence-producing components.

**Conclusions:**

People's concerns about excessive flatulence from eating beans may be exaggerated. Public health nutritionists should address the potential for gastrointestinal discomfort when increasing fiber intake from beans with clients. It is important to recognize there is individual variation in response to different bean types.

## Background

The 2010 Dietary Guidelines for Americans (DGA) emphasizes the benefits of a plant-based diet for better health. These recommendations include consumption of legumes, such as beans, several times per week [1]. Although consumers do recognize beans as a protein source or meat substitute, many overlook the fact that, like other vegetables, beans are rich sources of fiber, vitamins and minerals. Increasing bean consumption is a convenient and inexpensive way to enhance vegetable intake, as well as boost satiety of meals. Unfortunately, many consumers avoid eating beans, such as pinto, black, and kidney, because they fear that excessive intestinal gas or flatulence may result [2,3].

Increased flatulence is an expected outcome among some people when dietary fiber intakes are greater. This is particularly true if people already have low fiber intake. Traditional advice includes the belief that the body will adjust to the added fiber if regular legume consumption continues. The sensation of increased gas does seem to modulate with more frequent legume consumption [4]. Although for some people, just the expectation of excessive flatulence from eating beans may influence their perceptions of having gas [4,5].

### Dietary fiber and beans

Beans are naturally high in fiber, a food component that has been associated with lowered cholesterol concentrations, improved gastrointestinal (GI) function, and overall reduction of chronic disease risk [1]. Fiber may slow the absorption of carbohydrates, thus reducing possible hyperglycemia and serum insulin levels from carbohydrate-rich foods and increasing insulin sensitivity [6]. Fiber speeds digestive transit time by adding to fecal bulk [7]. Fiber intake also serves as a marker for fruit and vegetable consumption.

Normal intestinal function eludes many individuals [8,9]. Constipation and irritable bowel syndrome are common complaints [10]. For a variety of reasons, people may use fiber supplements instead of relying on fiber from whole foods such as beans to help lessen their symptoms. Chronic use of fiber supplements may have an adverse effect on gut health by leading to increased cell proliferation [11]. Because natural fiber sources like beans, such as pinto, black, or kidney, are preferable to artificial supplements, their consumption should be encouraged, but consumers' concerns about increased flatulence must be addressed [2,12].

### Why beans may cause intestinal gas

Most legumes contain relatively high amounts of both dietary fiber and resistant starches. The soluble oligosaccharides found in legumes are not digestible by human intestinal enzymes alone. Instead, oligosaccharides such as raffinose and stachyose are broken down by bacterial fermentation in the intestines [13]. Although some rectal gas is due to the ingestion of air, the majority of flatulence is produced from bacterial fermentation [14]. The byproducts of this degradation are hydrogen, carbon dioxide, methane, and sometimes sulfur, depending upon the bacteria. Normal intestinal processes move these gases out of the body in the form of flatus [15]. Removal or alteration of the oligosaccharide content of legumes will reduce the amount of gas produced [16,17]. However, it is not clear if changing the oligosaccharide component will alter the health benefits of legumes.

### Variations in gas production

There is variability among individuals in terms of intestinal gas production [15,18]. Some of the diversity may be due to differences in the types of microflora in the intestine, but further investigation of this topic is needed. Most healthy people adapt to fluctuations in GI gas production. In fact, bean consumption is substantially higher in many other countries than in the United States. Populations in Eastern and Southern African countries annually consume up to 50 kg or 110 pounds of beans per capita [19]. In contrast, annual per capita bean consumption in the United States has been estimated at 7.2 pounds [20].

The normally functioning digestive system moves gas through the intestine for expulsion. However, individuals who may have symptoms characteristic of irritable bowel syndrome (IBS) or other unexplained symptoms may experience intestinal gas pooling, regardless of whether or not there is an actual increase in intestinal gas production [21]. Impaired propulsion of gas through the digestive tract may result in bloating in the absence of actual increased gas production [22]. Hence, different physiological mechanisms produce flatulence and bloating.

The current study assesses self-reported data on GI symptoms among adults after eating ½ cup of beans or a control food product daily over 8 or 12 weeks. Participants were part of three separate, but similar, clinical trials designed to investigate the effect of daily bean consumption on biomarkers of heart disease risk [23-25]. All three studies showed significant reductions in total cholesterol and low-density-lipoprotein cholesterol concentrations in comparison to control foods over the 8 or 12 week intervention periods. A secondary objective of these three studies was to monitor GI symptoms and acceptability of the bean interventions. Differences in GI responses between the types of beans consumed were evaluated. These research projects were not designed to test the amount of flatus or hydrogen gas produced by consumption of the bean varieties. The few other studies that have looked at the acute effects of bean consumption such as pinto, black, or kidney on GI symptoms have been in metabolic ward settings, had participants consume beans for only a few days, or used processed bean powders instead of whole foods [3,15,26].

## Methods

### GI Questionnaire

The investigators developed a questionnaire to assess GI discomfort issues after daily consumption of ½ cup of beans. The questionnaire was based upon concerns (such as increased flatulence, changes in stool, and bloating) expressed by consumers about eating beans in other research studies [2-4]. The structure and content were modeled after a quality of life questionnaire validated for people with functional digestive disorders by Chassany et al. [27]. For our study, the questionnaire time frame was shortened from two to one week of recall. A series of 4 closed-ended yes/no questions ascertained if a person experienced changes in flatus frequency, stool frequency, stool consistency, or bloating frequency following daily bean consumption in the past week. Those who reported a change were asked to indicate the direction of the change (increase or decrease), the magnitude of these changes on an ordinal scale from 1 (*least change*) to 5 (*greatest change*), and if these changes had caused them to alter their social activities or daily routine. Responses were collected each week for the previous 7-day period. The GI questionnaire was reviewed for content validity by seven registered dietitians with clinical experience in digestive health. It was subsequently pilot tested with 12 adults for clarity of wording and response categories. After piloting, the questionnaire underwent minor revisions in wording and question order for more efficient administration prior to being used in the three studies. In addition, the instrument inquired about daily compliance with the research protocol and if the participants had eaten any other legume products including soy. The questionnaire is available by request from the corresponding author (DMW).

### Study designs

In Studies 1 and 2, participants were free to eat the daily ½ cup of beans served plain or as part of a recipe. Study 1 or the pinto and black-eyed pea (BEP) study (Pinto/BEP) was a 3 × 3 randomized cross-over trial designed to investigate the effects of ½ cup of pinto beans (*Phaseolus vulgaris*), black-eyed peas (*Vigna unguiculata)*, or canned carrots (as a control) on biochemical markers for heart disease and type 2 diabetes. These biomarkers included lipids, glucose, insulin, and C-reactive protein. Volunteers with a fasting insulin level ≥ 15 μU/ml were eligible to participate because one of the original study objectives was to determine the effect of the bean types on insulin levels of individuals with mild to moderate insulin resistance. Seventeen out of 23 originally enrolled individuals completed the Pinto/BEP study [23].

Study 2 or the baked bean study (BB) was similar to the Pinto/BEP study but utilized a 2 × 2 randomized cross-over design to investigate the effects of ½ cup of vegetarian baked beans made with navy beans (*Phaseolus vulgaris*) or canned carrots (control) on the same biomarkers as in the Pinto/BEP. A fasting total cholesterol concentration of 200 mg/dL or greater was required to be eligible for the BB study. Twenty-nine out of 33 initially enrolled individuals completed the BB study. Participants ate each food item for 8 weeks in both Pinto/BEP and BB studies. Participants were asked to not eat any other legumes including soy besides the ½ cup of canned beans or carrots over the course of the studies. The randomized 8 week treatment phases were followed by a minimal 2-week washout period in between. Full details and results of these studies are reported elsewhere [23,24].

In both the Pinto/BEP and BB studies, participants were provided multiple copies of the GI questionnaire at study entry and instructed to monitor their symptoms daily over the course of the week. They completed the GI questionnaire in-person on even weeks when they came to the study site to pick up additional food products. On odd weeks, participants completed the GI questionnaire as part of a regularly scheduled telephone interview to monitor protocol adherence and answer any questions they might have.

The third study was the pinto bean parallel-arm study or PintoPA designed to investigate the effects of pinto bean consumption on *in vitro *fecal bacterial fermentation, the production of short chain fatty acids, types of bacteria species in the gut, and serum lipid profiles [25]. A total of 80 adults completed the 12-week study. Each day, as part of their normal diet, half of the study group was fed an entrée containing a ½ cup of canned pinto beans (*P. vulgaris*), while the other half received control meals including a variety of chicken soups with similar caloric values as the bean entrées. The habitual diets of these free-living individuals were not altered in any way beyond consumption of the dietary treatments. Participants were asked to not eat other legume or soy-containing foods during the study. Both the treatment and control meals were prepared as prepackaged frozen entrées at the study site. Participants were instructed to eat them as provided without alteration. Participants in the PintoPA study completed the GI questionnaire each week and turned it into investigators at their weekly visit. The Institutional Review Boards at both study locations approved all aspects of studies Pinto/BEP, BB and PintoPA, and participants gave informed written consent prior to enrollment.

### Dietary intakes

For cross-over trial studies Pinto/BEP and BB, participants completed 24-hour recalls for a minimum of 2 days at baseline or the start of the two trials, and for 4-5 randomized days during each 8-week phase of the interventions [23,24]. Dietary records were entered into the Food Processor software for analysis (v. 8.4, ESHA Research, Salem, OR) and averaged for each study phase. In the PintoPA study, participants completed a consecutive 3-day diet record for two weekdays and one weekend day before the start and at the end of the intervention period. Diet records for the PintoPA study were analyzed using the Grand Forks Research Analysis of Nutrient Data software, which is a combination of the USDA Nutrient Database for Standard Reference and direct nutrient analysis of foods conducted by the researchers at the study site [25].

### Statistical analysis

Data from all GI questionnaires were examined for completeness and coding accuracy. A total of five participants in studies Pinto/BEP and BB missed one week each in one phase of the studies during weeks 3-8. Three people in weeks 10-12 of the PintoPA trial did not complete their questionnaires. Responses were otherwise complete for all cases. The main variables for analysis were bean type (pinto, black-eyed pea, and vegetarian baked bean), study type for the pinto bean treatments (cross-over vs. parallel arm), gender, participant age, and types of GI symptoms experienced. Dichotomous variables were created for presence or absence of changes in flatulence, stool, and bloating for each week of the study. Reports self-attributed by the participants to illness or medication adjustments (*n *= 7) were not counted as a change in our analyses.

For the two cross-over trials (Pinto/BEP and BB), a summary variable was created after an examination of these frequency distributions over the two 8 week studies. First, the weekly reports for flatulence, stool change, and bloating were examined and classified as 0, 1, 2, or 3 symptoms for each week. Based on these weekly observations, a variable was constructed to reflect the overall magnitude of reported GI difficulties for each of the bean types and control. The variable was coded 1 for zero to one reported symptoms or changes, 2 for moderate symptoms defined as two to four reports of increased flatus, stool change, or bloating, and 3 for excessive symptoms defined as five or more reports of increased flatus, loose stools, or bloating over each 8 week intervention phase. The same procedures were done for the PintoPA study with summary variables generated for those who ate the pinto bean meals and those who consumed the control foods for 12 weeks.

The SPSS Statistics software version 18.0 (IBM Corporation, Somers, NY) was used for all data analysis from the three GI questionnaires. Reports of symptoms were analyzed by gender, age, and body mass index (BMI) as a proxy for body size using ANOVA or Chi-square as appropriate to detect differences in the means of participant age by GI symptom categories within each bean type. Data are presented as the mean ± standard deviation (SD) unless otherwise noted. Statistical significance was indicated by a *p *value of ≤0.05.

## Results

Data are presented on participants from the two randomized cross-over trials (Pinto/BEP and BB) and one 2 × 2 factorial parallel arm trial (PintoPA). There were no statistically significant differences between the study-group characteristics (Table 1). Almost all of the participants in the three trials self-identified as white with a mean age of 42 years, and an average BMI of 28.3 kg/m^2 ^(Table 1).

**Table 1:** Baseline demographic characteristics of study participants in an investigation of perceptions of flatulence from beans

	Cross-over trials		Parallel arm trial	
	
	3 × 3 cross-over Pinto/BEP Pinto/Black eye/Control	2 × 2 cross-over BB Baked bean/Control	PBPA Pinto bean	Soup (control)
n	17	29	40	40

Male	41% (7)	41% (12)	50% (20)	50% (20)
Female Race Hispanic	59% (10) 94% White (16) 18% (3)	59% (17) 83% White (24) 7% (20)	50% (20) 100% White (40) n/a	50% (20) 97.5% White (39) n/a
Age, years	43.3 ± 12.6	44.9 ± 11.5	40.7 ± 10.0	39.2 ± 12.0
BMI, kg/m^2^	28.7 ± 5.1	26.5 ± 4.7	26.0 ± 3.0	31.9 ± 3.4

^a ^Values are mean ± SD.

### Studies Pinto/BEP and BB - cross-over trials

In the first week of each bean intervention of the randomized cross-over trials, reports of increased flatulence varied by bean type (Table 2). The fewest accounts of increased flatulence occurred with the black-eyed peas (19%). About the same number of people had increased flatulence with the pinto (50%) and vegetarian baked beans (47%). Only one person had increased flatulence with the carrot control treatment in week 1. By the second week of the cross-over trials, reports of increased flatulence dropped to 6% of participants for the pinto beans, 12% for the black-eyed peas and to 24% for the vegetarian baked beans. Reports of increased flatulence continued to decline over weeks 3-8, with only one to two participants reporting these symptoms with the pinto beans or the black-eyed peas. For the cohort in Study BB, the percentage of persons reporting increased flatulence stayed at 29% in week 3, but dropped to 11% in week 4. Two to three or 7-11% of participants continued to report increased flatulence with the baked beans during weeks 4-8. One to four participants reported increased flatulence while consuming the canned carrots during the control phases of both the Pinto/BEP and BB studies in weeks 1-4, but this reporting ceased in weeks 5-8 (Table 2). An increase in flatulence from the carrots was not expected, as their dietary fiber is less than that of the bean varieties.

**Table 2:** Percentage of cross-over trial participants reporting increased flatulence each week by food type

	Flatulence Change	Cross-over Trials			
		**BEP Study**		**BB Study**	
**Week #**	**All Beans** **% (n)**	**Pinto beans** **(n = 17)**	**Black eyed peas** **(n = 17)**	**Vegetarian** **Baked beans** **(n = 29)**	Control (carrots) (n = 39*)

1	35% (26)	50% (8)	19% (3)	47% (14)	3% (1)
2	19% (14)	6% (1)	12% (2)	24% (7)	11% (4)
3	15% (11)	0	6% (1)	29% (8)	5% (2)
4	11% (8)	12% (2)	6% (1)	11% (3)	5% (2)
5	5% (4)	6% (1)	0	11% (3)	0
6	5% (4)	6% (1)	6% (1)	7% (2)	0
7	5% (4)	0	6% (1)	11% (3)	0
8	3% (3)	6% (1)	6% (1)	7% (2)	0
*X*^2 ^P value	<0.001	<0.001	0.653	<0.001	0.075

*Seven people participated in both cross-over trials and thus completed only one control phase.

Reports of stool change in frequency or consistency were fewer than for flatulence. For all beans in the Pinto/BEP and BB studies, only 10% of participants reported an increase in stool frequency the first week. Twenty-four percent of the participants eating pinto beans and 10% of those eating vegetarian baked beans reported a change. One to two people reported changes in stool frequency or consistency for black-eyed peas and carrots. Throughout the remaining weeks of the two cross-over studies, only one to two people reported a change in stool frequency. A similar situation was observed for reported bloating, with those participants consuming the pinto beans experiencing the greatest bloating increase in the first week (29%), followed by those eating vegetarian baked beans (14%), and last by those eating carrots (8%) and black-eyed peas (6%) (Table 3). Mean ages and BMIs were not significantly different by reported symptoms or lack of symptoms within and across genders. However, women reported stool change more often than men (4.7% vs. 0.3%; *p *= 0.000).

**Table 3:** Percentage of participants reporting increased bloating frequency during each study week by food item consumed for cross-over trials.

	Bloating Increase	Cross-over Trials			
		**BEP Study**		**BB Study**	
**Week #**	**All Reports** **(n = 102)**	**Pinto beans** **(n = 17)**	**Black eyed peas (n = 17)**	**Vegetarian** **Baked beans** **(n = 29)**	Control (carrots) (n = 39*)

1	13% (13)	29% (5)	6% (1)	14% (4)	8% (3)
2	13% (13)	6% (1)	24% (4)	21% (6)	5% (2)
3	7% (7)	12% (2)	12% (2)	7% (2)	3% (1)
4	4% (4)	6% (1)	0	7% (2)	3% (1)
5	5% (5)	6% (1)	6% (1)	10% (3)	0
6	6% (6)	6% (1)	6% (1)	10% (3)	3% (1)
7	3% (3)	0	0	7% (2)	3% (1)
8	2% (2)	0	6% (1)	0	3% (1)
*X*^2 ^p value	0.009	0.043	0.204	0.403	0.747

*Seven people participated in both cross-over trials and thus completed only one control phase.

**Figure 1:**
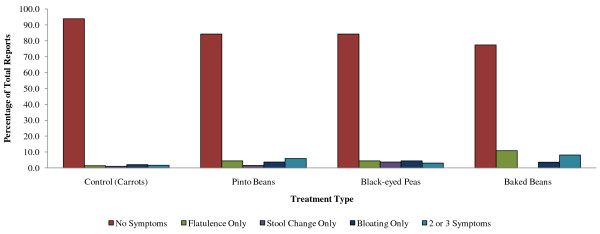
**Percentage of all reports indicating gastrointestinal symptoms by food type in two randomized cross-over studies**.

For the overall GI symptom variable in the Pinto/BEP study, most participants (88%, *n *= 15 for pinto beans and 82%, *n *= 14 for black-eyed peas) reported zero or only one incidence of increased flatulence, stool change or bloating over each 8-week period. Twelve percent (*n *= 2) and 18% (*n *= 3) respectively reported two to four incidences of GI discomfort for these two food interventions, but none of the participants reported excessive symptoms, defined as more than five reports of a problem over each 8 week intervention phase. Seventy-two percent (*n *= 18) of the participants in the BB study reported zero or only one symptom of GI discomfort over the 8-week study period, 16% (*n *= 4) reported two to four symptoms and 12% (*n *= 3) reported excessive GI symptoms. All three participants who had excessive GI symptoms with the BB study were men. However, no significant differences were found for the categorical summary variable of symptom reporting with regard to gender, bean type, BMI, or macronutrient intakes, including dietary fiber (Figure 1).

Dietary intake data obtained from the 24 hour recalls during the cross-over trials Pinto/BEP and BB are shown in Table 4 for each of the baseline, intervention and control phases. There were no significant differences in nutrient intakes with the exception of dietary fiber between the vegetarian baked beans and baseline phases. The fiber content of the pinto beans and the vegetarian baked beans was 7 grams per ½ cup serving. The black-eyed peas contained 4 grams, and the carrots contained 2 grams per ½ cup serving.

**Table 4:** Mean nutrient composition of cross-over trial participant's 24-hour dietary recalls during treatment and baseline phases (n = number of food records per phase)

	Pinto/BEP Study		BB Study		
**Nutrients** **(n = # of food records)**	**Diet + Pinto Beans (n = 101)**	**Diet + Black Eyed Peas (n = 101)**	**Diet + Baked Beans (n = 101)**	**Diet + Control Carrots (n = 102)**	**Baseline** **(n = 67)**

Energy (kcal/d)	2078 ± 148	2128 ± 184	2082 ± 114	2112 ± 111	1931 ± 87
Protein (g/d)	79 ± 5	87 ± 9	87 ± 5	86 ± 5	79 ± 4
Carbohydrates (g/d)	265 ± 15	275 ± 19	265 ± 16	264 ± 14	245 ± 12
Dietary Fiber (g/d)	23 ± 1.6	20 ± 1.6	*26 ± 1.5	21 ± 1.3	*19 ± 1.0
Total Fat (g/d)	75 ± 6	77 ± 9	73 ± 6	79 ± 5	71 ± 4
Cholesterol (mg/d)	213 ± 19	253 ± 28	269 ± 34	262 ± 23	244 ± 26
Sodium (mg/d)	2951 ± 141	3377 ± 296	2868 ± 246	3196 ± 206	3116 ± 204

Data are presented as means ± SEM. *Dietary fiber mean is significantly different, *P*<.05.

### Study 3 - PintoPA

**Figure 2:**
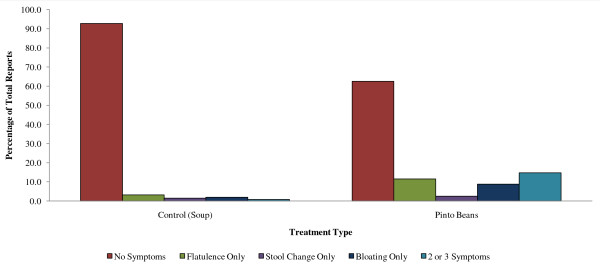
**Percentage of all reports indicating gastrointestinal symptoms by food type in parallel arm trial**.

Similar results were observed for pinto beans and flatulence in the PintoPA trial as were seen in the Pinto/BEP cross-over study. Forty-five percent reported increased flatulence with pinto bean consumption during the first week of the study. However, the reported percentage dropped to 38% in the second week and to 30% by the third week. For weeks 6-12, 15-23% continued to report increased flatulence. The canned pinto beans utilized in this trial were identical to those in cross-over study Pinto/BEP. Participants in the control arm of the trial consumed a soup that did not have any known flatulence-producing ingredients. Three to eight percent of control arm participants consistently reported increased flatulence throughout the 12 weeks of the trial (Table 5). This rate is similar to that seen for the canned carrots control food in cross-over studies Pinto/BEP and BB. Eight to 20% of the subjects consuming pinto beans reported stool changes across all 12 weeks of the PintoPA study, while1-2 people reported stool changes on the control soup diet. Reports of increased bloating frequency were 25% for the first week, and 40% for the second week for those consuming the pinto bean treatment (Table 6). Bloating continued to be reported by 3-5% of the control arm group throughout the 12-week study (Figure 2). Dietary intake data from 3-day food records for the participants in the intervention and control arms of the trial are shown in Table 7. There were no significant differences in intakes between cohorts with the exception of fiber for the control group.

**Table 5:** Percentage of parallel arm trial participants reporting increased flatulence each week by food type

	PintoPA Trial	
Week #	Pinto beans (n = 40)	Control soup (n = 40)
1	45% (18)	0
2	38% (15)	8% (3)
3	30% (12)	5% (2)
4	23% (9)	5% (2)
5	30% (12)	3% (1)
6	15% (6)	3% (1)
7	20% (8)	8% (3)
8	23% (9)	3% (1)
9	20% (8)	3% (1)
10	15% (6)	0
11	15% (6)	5% (2)
12	20% (8)	8% (3)
*X*^2 ^P value	0.034	0.660

**Table 6:** Percentage of parallel arm trial participants reporting increased bloating frequency each week by food type

	PintoPA Trial	
Week #	Pinto beans (n = 40)	Control soup (n = 40)
1	25% (10)	5% (2)
2	40% (16)	0
3	15% (6)	3% (1)
4	25% (10)	0
5	20% (8)	3% (1)
6	28% (11)	3% (1)
7	18% (7)	5% (2)
8	10% (4)	3% (1)
9	10% (4)	3% (1)
10	23% (9)	3% (1)
11	15% (6)	0
12	15% (6)	5% (2)
*X*^2 ^P value	0.142	0.731

**Table 7:** Mean nutrient composition of parallel arm participant's 3-day food records during intervention and baseline phases

	PintoPA Trial			
**Nutrients** **(n = # of food records)**	**Baseline Diet Before Beans (n = 114)**	**Exit Diet + Pinto Beans (n = 114)**	**Baseline Diet Before Control (n = 120)**	**Baseline Diet + Control Soup** **(n = 120)**

Energy (kcal/d)	2078 ± 148	2128 ± 184	2082 ± 114	1931 ± 87
Protein (g/d)	79 ± 5	87 ± 9	87 ± 5	79 ± 4
Carbohydrates (g/d)	265 ± 15	275 ± 19	265 ± 16	245 ± 12
Dietary Fiber (g/d)	23 ± 1.6	20 ± 1.6	*26 ± 1.5	*19 ± 1.0
Total Fat (g/d)	75 ± 6	77 ± 9	73 ± 6	71 ± 4
Cholesterol (mg/d)	213 ± 19	253 ± 28	269 ± 34	244 ± 26
Sodium (mg/d)	2951 ± 141	3377 ± 296	2868 ± 246	3116 ± 204

Data are presented as means ± SEM. *Dietary fiber mean is significantly different, *P*<.05.

Reports of GI symptoms were analyzed by gender and age for the pinto beans and the control soup. Mean ages were not significantly different by reported symptoms or lack of symptoms within and across genders. For the participants in the pinto bean trial arm, there were significant differences between women and men with regard to reported symptoms. In these data, women reported bloating (13.1% vs. 4.6%) and stool change (3.4% vs. 1.7%) more often than men (*p *= 0.000).

## Discussion

### Differences in bean varieties on flatulence production

Studies investigating actual production of flatus have typically used beans from the genera *Phaseolus *such as Great Northern [18], California small white beans [28], or red kidney beans [3,29]. More recent investigations have assumed the flatulence potential of legumes based on their nutrient profile (black-eyed peas) or objective observations by participants (soybeans) rather than direct quantification of flatus with human subjects [30,31]. Little has been written about the impact on gas production of other varieties of beans or on beans eaten for longer than a few days or weeks.

Our study described the perceived amount of GI discomfort experienced by people from regular bean consumption over several weeks. The results are consistent from the two cross-over studies and the parallel arm study, indicating that although increased flatulence may occur in some individuals with regular (i.e., daily) bean intake of ½ cup, not all people are affected. The research findings are unique in that only half or fewer of the participants experienced the sensation of having more gas in the first week of diet change. Seventy percent or more of the participants who experienced flatulence felt that it dissipated by the second or third week of bean consumption. The black-eyed peas with lower fiber content elicited less of a response in most people in comparison to the pinto and navy beans with higher fiber content. However, fiber content alone does not explain the observed differences in perception of flatulence and GI symptoms across the studies. A small, but consistent percentage of participants (3-11%) reported increased flatulence even when fed control diets that did not contain known flatulence-producing components. However, no consistent predictors of persistent increased flatulence such as gender, age or BMI were found. To judge from these findings, much of the concern by the public about excessive flatulence from eating beans may be exaggerated. However, public health professionals and dietitians should be candid with their clients and address the potential for GI discomfort when increasing bean and fiber intake.

There have been few studies investigating the perceived changes in GI function from eating beans. The documentation of variability in these perceived effects based on three different types of beans is a strong point for promoting different types of legumes in the diet. Since the study did not quantitatively determine the amount of flatus or gas produced, our findings are limited to the subjective reports of our participants.

### Psychological anticipation of flatulence problems

Increased amounts of fermentable dietary fiber in beans will increase the production of intestinal gas by bacterial flora. However, individual effects of this increased gas production may vary from being partially reabsorbed, to being expressed without discomfort, to being repressed due to the noxious odor or volume [14]. It is possible that if people believe that eating beans will cause flatulence they will perceive an increase in symptoms. The fat substitute olestra provides an example of the power of the mind to influence perceptions of symptoms. Because foods containing olestra may cause increases in cramping, diarrhea, and flatulence, Sandler et al. conducted a randomized case-control study to investigate the effects of olestra on GI symptoms [32]. All participants received information regarding these symptoms, and the control product was labeled as containing olestra. Although the latter group received no olestra, they still reported increases in GI symptoms [32]. It is possible, as shown in the olestra experiment, that some individuals perceive digestive changes from eating beans regardless of the magnitude of the real effects [5,32].

## Conclusions

Our findings have several practical applications within the fields of nutrition and public health. First, perception of flatulence increase is variable by bean type and across individuals. Second, after a few weeks of daily bean consumption, people perceive that flatulence occurrence returns to normal levels. Third, a small percentage of individuals may be bothered by increased flatulence regardless of the length of time they consume legumes.

To help the public incorporate more beans into their diets, the potential of increased flatulence should be addressed. People can be made aware that increasing beans in the diet may result in more flatulence initially. However, clinicians are in a good position to emphasize that the flatulence will decrease over time if bean consumption is continued and that the nutritional attributes of beans in the diet outweighs the potential for transitory discomfort. The long-term health benefits of bean consumption are great. Stressing these health promotion aspects to consumers, as well as imparting practical knowledge that perceived increases in flatulence are most likely temporary, can go far in persuading consumers to add more beans to their diet. Some helpful suggestions might be to initially eat smaller portions of beans or to divide servings of beans in half. Consumers should also evaluate different bean varieties to determine if certain types produce greater desirable or undesirable symptoms than others.

## Abbreviations

ANOVA: Analysis of variance; BB: Baked beans; BEP: Black-eyed peas; BMI: Body mass index; DGA: Dietary Guidelines for Americans; GI: Gastrointestinal; IBS: Irritable bowel syndrome PintoPA: Pinto bean parallel arm trial.

## Competing interests

The authors declare that they have no competing interests.

## Authors' contributions

DMW and AMH designed the study together. DMW designed and pilot-tested the questionnaire, and drafted the manuscript. AMH assisted with statistical analysis and manuscript revision. Both authors read and approved the final manuscript.
